# Metastatic EML4-ALK fusion detected by circulating DNA genotyping in an EGFR-mutated NSCLC patient and successful management by adding ALK inhibitors: a case report

**DOI:** 10.1186/s12885-016-2088-5

**Published:** 2016-02-05

**Authors:** Wenhua Liang, Qihua He, Ying Chen, Shaokun Chuai, Weiqiang Yin, Wei Wang, Guilin Peng, Caicun Zhou, Jianxing He

**Affiliations:** Department of Thoracic Surgery and Oncology, The First Affiliated Hospital of Guangzhou Medical University, Guangzhou, China; Guangzhou Institute of Respiratory Disease & China State Key Laboratory of Respiratory Disease & National Clinical Research Center for Respiratory Disease, No. 151, Yanjiang Rd, Guangzhou, 510120 Guangdong Province PR China; Burning Rock Biotechnology Co. Ltd., Guangzhou, China; Department of Oncology, Shanghai Pulmonary Hospital, Tongji University School of Medicine, Shanghai, China

**Keywords:** NSCLC, EGFR mutation, EML4-ALK rearrangement, Co-existence

## Abstract

**Background:**

Rebiopsy is highly recommended to identify the mechanism of acquired resistance to EGFR-TKIs in advanced lung cancer. Recent advances in multiplex genotyping based on circulating tumor DNA (ctDNA) provide a strong and non-invasive alternative for detection of the resistance mechanism.

**Case presentation:**

Here we report a multiple metastatic NSCLC patient who was detected to have pure EGFR 19 exon deletion (negative for EML4-ALK and ROS1 in both IHC-based and sequencing assay) in the primary lesion and responded to first-line and second-line EGFR-TKI treatments (erlotinib then HY-15772). At 8 months, most lesions remained well controlled except for the liver metastases which presented dramatic progression. Considering the high risk of bleeding in rebiopsy of hepatic lesions, we conducted a multiplex genomic profiling with ctDNA. Results reported coexistence of EGFR mutation and EML4-ALK gene translocation in plasma which heavily indicated that ALK was the primary reason for progression of the liver lesions. This deduction was supported by the repeated response to ALK inhibitors (crizotinib then AP26113) of the hepatic metastases.

**Conclusions:**

This is the first report of the existence of ALK rearrangement in metastatic lesions in an EGFR mutated patient. It highlighted the feasibility and advantages of using ctDNA multiplex genotyping in identifying the heterogeneity across lesions and the resistance mechanism of targeted treatments.

**Electronic supplementary material:**

The online version of this article (doi:10.1186/s12885-016-2088-5) contains supplementary material, which is available to authorized users.

## Background

Advances in geno-typing have changed the clinical practice of treatment of non-small cell lung cancer (NSCLC), especially non-squamous types where driver mutations, e.g. epidermal growth factor receptor (EGFR) mutations and echinoderm microtubule-associated protein-like 4-anaplastic lymphoma kinase (EML4-ALK) translocation are commonly present. Agents that target EGFR activating mutations (gefitinib, erlotinib, and afatinib, etc.) or ALK rearrangement (crizotinib, etc.) derive significantly greater benefits than cytotoxic chemotherapy in patients who harbor these gene alterations, which is consistently proved by extensive large-scale randomized controlled trials [1, 2]. In order to deliver an appropriate first-line treatment regimen, detection of EGFR mutation and ALK rearrangement are recommended as routine genetic profiling for non-squamous NSCLC or non-smoking populations [3]. In recent years, some selective inhibitors that can overcome the resistance to first generation inhibitors of these driver alterations have also been developed, e.g., AZD9291 and CO-1686, some more effective inhibitors against both EGFR sensitizing and resistance T790M mutations [4]; or ceretinib, aletinib and AP26113, the agents that are effective for both ALK fusion and some secondary gatekeeper mutations [5].

Direct sequencing and amplification refractory mutation system (ARMS)-PCR are the common testing methods for EGFR mutations. FISH, RT-PCR and Ventana IHC are all currently accepted methods for detection of ALK rearrangement. In recent years, the development and advances in high throughput next-generation sequencing (NGS) have allowed the simultaneous profiling of alterations in multiple genes [6]. Circulating tumor DNA (ctDNA) is released or excreted by tumour cells and circulates in the blood of a cancer patient; analysis of the fraction of mutant-alleles from ctDNA compared to normal-alleles from the patients’ normal genome provides opportunities for minimally-invasive cancer diagnosis and tumor monitoring [7]. Detection with ctDNA, which originates from all potential lesions, could overcome the disadvantages of single site biopsy given that the intra-tumoral and inter-lesional heterogeneity is common [6, 8]. Enrichment of plasma ctDNA and incorporation with the next generation deep sequencing techniques allow us to simultaneously detect the gene alterations of interest, e.g. EGFR/BRAF/HER2 mutations, ALK/ROS1/RET rearrangements, MET amplification, etc. in NSCLC, especially when it is difficult to obtain sufficient tissue samples.

EGFR mutations and ALK rearrangement are generally considered to be mutually exclusive. However, some recent surveys and case reports showed co-existence of the two alterations within the same lesion [9, 10]. Here, we report an advanced NSCLC case with EGFR exon 19 deletion who experienced single-site progression in the liver after primary response to EGFR-TKI treatments and showed good response when adding crizotinib after the detection of ALK rearrangement signal through ctDNA. This special case highlights the feasibility and necessity of using ctDNA multiplex genomic profiling as an alternative approach in molecular diagnosis of NSCLC or in the exploration of the underlying mechanism in resistance to targeted therapies. Moreover, it encourages us to re-evaluate the heterogeneity across lesions of metastatic NSCLC.

## Case Presentation

**Fig. 1:**
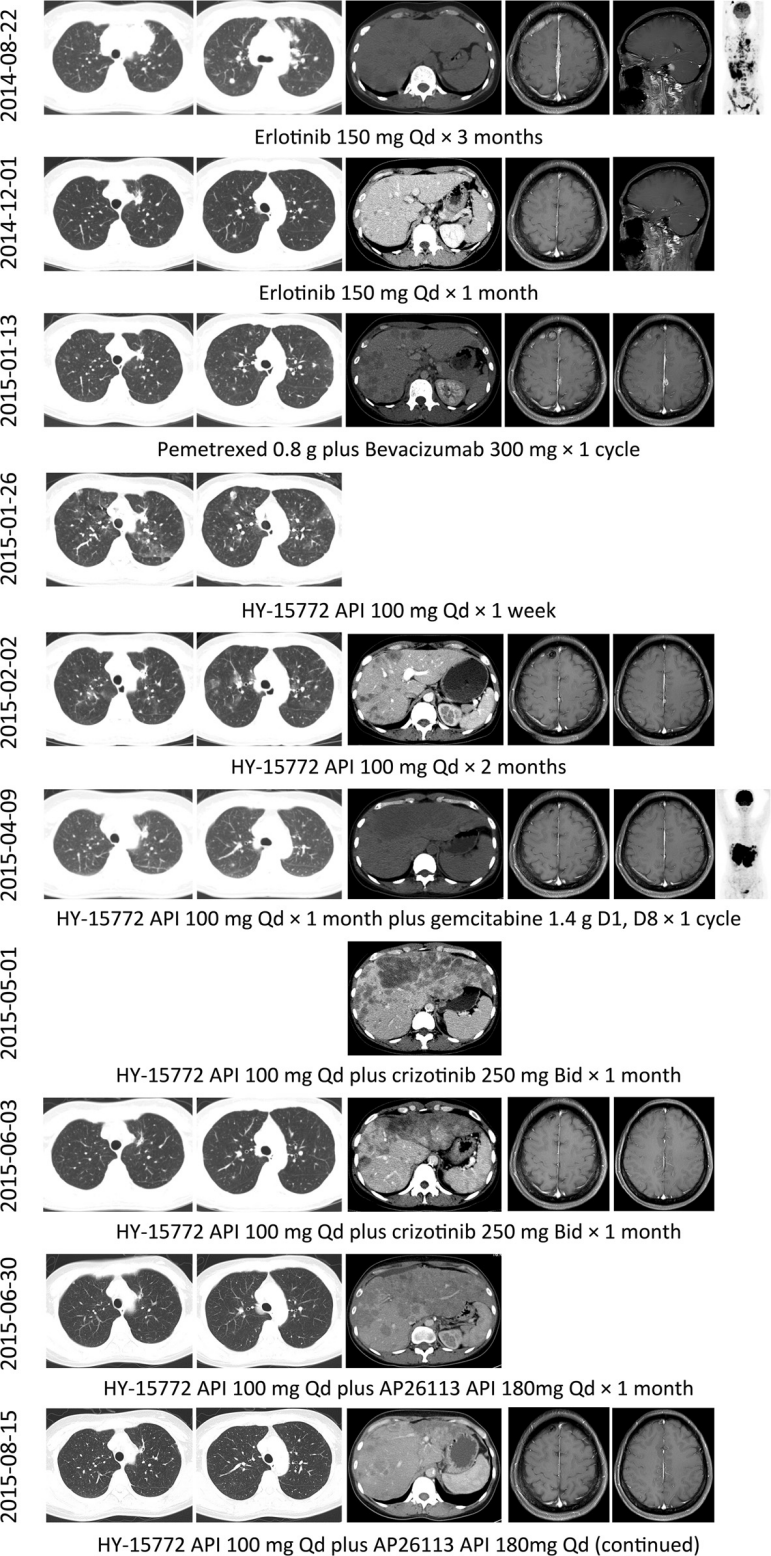
Changes in image during treatments. API, active pharmaceutical ingredient

A 46-year-old woman with stage IVb lung adenocarcinoma of the left upper lobe and extensive metastases (mediastinal lymph nodes, bilateral lung, liver, brain, multiple vertebrae, pelvis, adrenal glands, retroperitoneal lymph nodes, etc.) was confirmed to harbor EGFR 19 exon deletion by ARMS-PCR. She was negative for EML4-ALK and ROS1 by ventana IHC staining. Evaluation after 1 month and 3 months of erlotinib 150 mg Qd treatment showed good partial response across all lesions. However, the patient presented with severe shortness of breath after 4 months. CT scan showed rapid progression of the distributed pulmonary and hepatic lesions. Based on the imaging features and clinical symptoms, it was initially difficult to differentiate whether the patient had interstitial pneumonia due to erlotinib, or lymphangitis carcinomatosa. Thus, we firstly withdrew erlotinib and delivered methylprednisolone pulse therapy (500 mg qd * 5 days). However, no improvement was observed, which lead us to the diagnosis of lymphangitis carcinomatosa due to disease progression. Pemetrexed 0.8 g plus bevacizumab 300 mg (the tumor board decided to use a platinum-free regimen due to low tolerance to platinum of this patient, ECOG performance score 2–3), was then administered, but the lesions continued to grow rapidly. Noninvasive positive pressure ventilation was applied when the patient experienced type I respiratory failure. The patient took HY-15772, an active pharmaceutical ingredient (API) that was known for AZD9291, 100 mg qd on her own volition. She recovered from hyoxemia gradually and CT review showed tumor remission at 1 week after starting HY-15772 API. The patient continued to take HY-15772 API for 2 months. CT-scan showed that all lesions were controlled except for the liver metastases in the left lobe which underwent dramatic bulky progression. Multidisciplinary consultation suggested that a needle biopsy of the liver lesions was not preferable due to the high risk of procedure-related bleeding. Thus, we decided to obtain peripheral blood for ctDNA multiplex genotyping analysis using the capture probe baits sequencing platform (methods and gene list were provided in Additional file 1). Meanwhile, a dose of gemcitabine chemotherapy 1.4 g on day 1 and day 8 was administered. During the intermission, the ctDNA analysis reported the existence of both EGFR 19 exon L747S non-shifting deletion (abundance 48.5 %) and EML4-ALK rearrangement (abundance 10.09 %) in the plasma (No other druggable alteration was found especially in domains of MET). Upon the second cycle the gemcitabine chemotherapy, the patient complained of an obvious sensation of mass in the upper abdomen and the inability to keep food or drink down. We suggested the patient add crizotinib and continue HY-15772 API. After 5 days of additional crizotinib treatment, the patient was able to drink and eat and had the sensation of mass shrinkage. CT scan after 1 month reavealed significant remission of the lesions in the left lobe of the liver (plasma ALK abundance decreased to 2.79 %). After 2 months of crizotinib, liver metastases especially those in the left lobe again progressed, which was manifested as abdominal distention and edema of bilareal lower extremity (plasma ALK abundance increased to 14.59 %). The patient switched the ALK inhibitor to AP26113 API at a dose of 180 mg Qd on her own volition and quickly regained good remission of the hepatic lesions (plasma ALK abundance decreased to 0.00 %). In addition, plasma ALK fusion was not detected. Figure 1 illustrated the change of the image presentation and treatments. Upon submission of this report, the patient is still receiving a combination treatment of HY-15772 and AP26113 API without any signs of disease worsening. Re-analysis of the biopsy tissue from the primary lesion through the same multiplex genomic platform showed pure EGFR exon 19 deletion without any signal for EML4-ALK fusion. In addition, repeated analyses indicated the presence of TP53 Y236D mutations in 7 exon with more than 50 % abundance. Figure 1 illustrated the flow of treatments and image evaluation.

## Discussions

The current recommendation for management of single-site regional progression in resistance to EGFR-TKIs is to perform localized therapies and rebiopsy when available [11]. In this case, the progressed liver metastases were large, bulky, and scattered. Radiotherapy or radiofrequency ablation was not an appropriate option. Metastatic hepatic lesions were supplied by portal vein system rather than hepatic artery system. Thus, transcatheter arterial chemoembolization (TACE) might not derive significant benefits. On the other hand, this case progressed dramatically after showing resistance to EGFR-TKIs. In this case, switching to chemotherapy is suggested according to what IMPRESS trial indicated [12]. Considering the previous evidence of the patients’ insensitivity to pemetrexed, we decided to use gemcitabine single agent chemotherapy. Meanwhile, we continued the use of HY-15772 API since all extra-hepatic lesions remained stable or even undetectable in the images, which suggested these lesions remained sensitive to HY-15772 API.

Rebiopsy was another issue worth clarifying. In this case, as the entire left lobe of the liver was occupied by metastatic lesions that were loosened in structure, multidisciplinary consultation opinions suggested that conducting a fine-needle biopsy would be dangerous due to the high risk of bleeding. Recent advances in non-invasive rebiopsy approaches such as multiplex genomic profiling of ctDNA provided us an alternative [13]. We decided to obtain peripheral blood from this patient to perform a NSCLC-related multiplex genotyping, in order to clarify the resistance mechanism. Surprisingly, in addition to the 19del EGFR mutation that was previously discovered in the primary lesion, EML4-ALK fusion with relatively high abundance in plasma was detected. Given that the extra-hepatic lesions were all under control, indicating their lasting response to HY-15772 API, we added Crizotinib, the only approved agent to inhibit ALK-driving tumors, to the regimen without withdrawing the EGFR suppressor. Previous reports suggested no significant superimposed toxicity in dual treatment with EGFR and ALK inhibitors [14]. In this case, the patient presented rapid relief without any grade III adverse events. Undoutedly, the timely addition of ALK inhibitors salvaged the patient as previous gemcitabine showed no efficacy.

Though some previous studies reported co-existence of EGFR and ALK in the same lesions, this is the first report showing ALK rearragement in distant metastatic lesions in a patient potentially harboring pure EGFR mutations in the primary lesion. Since rebiopsy for the liver metastases in the left lobe was not an option in this case, we had no direct evidence that the liver progression was driven by ALK fusion. However, the great abundance of EML4-ALK fusion signals in the plasma upon disease progression and the significant response after adding crizotinib (as well as the AP26113 API after resistance to crizotinib) strongly supported the existence of ALK fusion in the growing liver metastases. In addition, it was not likely that the primary lesion harbored concurrent EGFR mutation and ALK fusion because both routine ARMS and NGS re-analysis showed that no ALK fusion was detected and the pulmonary lesions were all in good control by HY-15772 API when the hepatic metastases progressed. Such heterogeneity might be more common than we have previously acknowledged, and might have a great impact on treatment strategy. In the past, we could only detect genetic alterations with site by site tissue samples. It was too difficult to obtain tissues from all lesions; multiplex genotyping with ctDNA might help address this issue. Thus, with the current technology, we should reevaluate the heterogeneity across different lesions and their incidence. Of course, the sensitivity of ctDNA examination and the substantial validation require further efforts.

We cannot determine whether the ALK fusion was inherent or if it was an evolvement after treatment. One possibility is the clonal selection mechanism, EML4-ALK fusion emerged when EGFR mutations were suppressed. However, in both the treatment with erlotinib and subsequent HY-15772 API, the hepatic lesions showed good response to these EGFR-TKIs, which suggested no or minimal ALK fusion at that point. But some reports have also revealed responsiveness to EGFR-TKIs in patients with ALK fusion or dual alterations [15]. Another potential reason is the inducement or de novo activation of EML4-ALK fusion after EGFR-TKI treatments since repeated analyses showed consistent high abundance (over 50 %) of TP53 mutation which heavily indicated to the presence of loss of heterozygosity (LOH) phenomena [16]. Genome instability might increase the risk for inducing other driver gene altrations. Further studies on the mechanism is warranted.

## Conclusions

In conclusion, this is the first report of the existence of ALK rearrangement in metastatic lesions in an EGFR mutated patient. It highlighted the feasibility and advantages of using ctDNA multiplex genotyping in identifying the resistance mechanism of targeted treatments. Further well designed studies on its true effectiveness and criteria for elegible patient selection are warranted. In addition, ctDNA multiplex genotyping could benifit us in studying the heterogeneity across lesions of lung cancer.

## Availability of data and materials

Any additional supporting data involving details of clinical and genetic analysis can be found in the electronic medical record system of the First Affiliated Hospital of Guangzhou Medical University and are available upon request from the corresponding author.

## Additional file

Additional file 1:
**Platform and Gene-list for the Multiplex Genotyping of Circulating Tumor DNA.** (DOC 45 kb)

## Abbreviation

EGFR: Epidermal growth factor receptor
EML4-ALK: Echinoderm microtubule-associated protein-like 4-anaplastic lymphoma kinase
IHC: Immunohistochemistry
TKI: Tyrosine kinase inhibitor
