# Supplementary material for: Metastatic EML4-ALK fusion detected by circulating DNA genotyping in an EGFR-mutated NSCLC patient and successful management by adding ALK inhibitors: a case report
Source: BMC Cancer. 2016 Feb 5;16:62. doi: 10.1186/s12885-016-2088-5 (PMC4744376; doi:10.1186/s12885-016-2088-5)
Supplement: Additional file 1: — Platform and Gene-list for the Multiplex Genotyping of Circulating Tumor DNA. (DOC 45 kb) [file 12885_2016_2088_MOESM1_ESM.doc]

**Additional file 1**

**Platform and Gene-list for the Multiplex Genotyping of Circulating Tumor DNA**

Capture probe baits were produced to cover 161kb human genomic loci from 510 target regions, including selected exons and introns from 168 genes (Table S1) using the Agilent eArray platform. DNA fragments hybridized by the probe baits were amplified and sequenced on NextSeq500 (Illumina, Inc., USA) with pair-end reads. Sequence data were then mapped to the human genome (hg19) using BWA aligner 0.7.10. Local alignment optimization and variant calling was performed using GATK 3.2. DNA translocation analysis was performed using both Tophat2 and Factera 1.4.3.

Table S1. Gene-list

| ACER2 | CD5L | CYP3A4 | FLT3 | LRRC2 | NTRK1 | PSG5 | SOX2 |
| --- | --- | --- | --- | --- | --- | --- | --- |
| ADAMTS12 | CDH18 | DHX9 | GALNT13 | LRRC7 | OBP2A | PTEN | SPOP |
| ADAMTS16 | CDK4 | DPP10 | GATA3 | LTBP1 | OCA2 | PYHIN1 | SPTA1 |
| ADAMTS20 | CDK6 | DPYD | GFRAL | MDGA2 | ODZ3 | RAD50 | STK11 |
| AK5 | CDKN2A | EGFR | GKN2 | MEK1 | OR2T4 | RB1 | TBX15 |
| AKR1B10 | CHEK1 | EIF3E | GRIA3 | MET | OR4A15 | REG1B | TERT |
| AKT1 | CHEK2 | EPB41L4B | GUCY1A3 | MLL3 | OR4C6 | RET | TGFBR2 |
| ALK | CNGB3 | ERBB2 | HCN1 | MRPL1 | OR5L2 | RNF43 | THSD7A |
| AMOT | CNOT4 | ERBB3 | HGF | MSH6 | OR6F1 | ROS1 | TIMD4 |
| ANKRD30A | CNTN5 | EXOC5 | HRAS | MTOR | PDE1C | RYR2 | TMEM132D |
| APC | CNTNAP5 | EYA4 | IDH1 | MYC | PDE4DIP | SAGE1 | TNN |
| ARID1A | COASY | F9 | IDH2 | MYH2 | PIK3CA | SCN7A | TNR |
| ASTN1 | COL19A1 | FAM135B | IL1RAPL1 | NAV3 | PIK3R1 | SETD2 | TP53 |
| ATP10B | COL25A1 | FAM5C | ITM2A | NFE2L2 | POLDIP2 | SI | TPTE |
| BRAF | COL5A2 | FBN2 | JAK1 | NLRP4 | POLR3B | SLC26A3 | TRIM58 |
| BRCA1 | CSMD1 | FBXW7 | KDM5A | NOTCH1 | POM121L12 | SLC4A10 | TRIML1 |
| BRCA2 | CSMD3 | FGF19 | KEAP1 | NOTCH2 | POTEG | SLC5A1 | TRPC5 |
| BTRC | CTNNA2 | FGF3 | KLHL1 | NOTCH4 | PPP2R1A | SLC6A5 | U2AF1 |
| CACNA1E | CTNNB1 | FGF4 | KRAS | NRAS | PRKDC | SMAD4 | UGT1A1 |
| CBL | CYP2C19 | FGFR1 | KRTAP5-5 | NRXN1 | PRSS1 | SNTG1 | UNC5D |
| CCND1 | CYP2D6 | FGFR3 | LRP1B | NTM | PSG2 | SORCS3 | VPS13A |
